# DNA Methylation in Autism Spectrum Disorders: Biomarker or Pharmacological Target?

**DOI:** 10.3390/brainsci14080737

**Published:** 2024-07-23

**Authors:** Hanieh Gholamalizadeh, Maedeh Amiri-Shahri, Fatemeh Rasouli, Arina Ansari, Vafa Baradaran Rahimi, Vahid Reza Askari

**Affiliations:** 1 Student Research Committee, Mashhad University of Medical Sciences, Mashhad 13131-99137, Iran; gholamah961@mums.ac.ir; 2 Faculty of Medicine, Mashhad University of Medical Sciences, Mashhad 91779-48564, Iran; 3 Student Research Committee, North Khorasan University of Medical Sciences, Bojnurd 94149-75516, Iran; m.amirishahri@nkums.ac.ir (M.A.-S.); f.rasouli@nkums.ac.ir (F.R.); arinaansari80@gmail.com (A.A.); 4 Faculty of Medicine, North Khorasan University of Medical Sciences, Bojnurd 94149-75516, Iran; 5 Department of Cardiovascular Diseases, Faculty of Medicine, Mashhad University of Medical Sciences, Mashhad 91779-48564, Iran; baradaranrv@mums.ac.ir; 6 Pharmacological Research Center of Medicinal Plants, Mashhad University of Medical Sciences, Mashhad 91779-48564, Iran

**Keywords:** autism spectrum disorders, DNA methylation, epigenetics, animal model, biomarkers

## Abstract

Autism spectrum disorder (ASD) is a group of heterogeneous neurodevelopmental disabilities with persistent impairments in cognition, communication, and social behavior. Although environmental factors play a role in ASD etiopathogenesis, a growing body of evidence indicates that ASD is highly inherited. In the last two decades, the dramatic rise in the prevalence of ASD has interested researchers to explore the etiologic role of epigenetic marking and incredibly abnormal DNA methylation. This review aimed to explain the current understanding of the association between changes in DNA methylation signatures and ASD in patients or animal models. We reviewed studies reporting alterations in DNA methylation at specific genes as well as epigenome-wide association studies (EWASs). Finally, we hypothesized that specific changes in DNA methylation patterns could be considered a potential biomarker for ASD diagnosis and prognosis and even a target for pharmacological intervention.

## 1. Introduction

Autism spectrum disorder (ASD), characterized by deficits in social communication and the presence of restricted, repetitive behaviors or interests, is a neurodevelopmental disorder affecting approximately 2.3% of 8-year-old children and 2.2% of adults in the United States [1,2]. The estimated prevalence of ASD in the United States has increased from 1.1% in 2008 to 2.3% in 2018, likely due to changes in diagnostic criteria, enhanced screening and diagnostic tools, and raised awareness among the public [1]. ASD appears to be one of the most debilitating neurodevelopmental disorders in the United States, and estimates put the annual cost at close to USD 250 billion, with the expense of caring for an individual with ASD over their lifetime falling somewhere between USD 1.5 and USD 2.5 million [3].

Noteworthy, there are currently no diagnostic biomarkers identified as specific to ASD. In the first two years of a child’s life, common early signs and symptoms of ASD include no response to the child’s name when called, no or limited use of gestures for communication, and lack of imaginative play [1]. In addition to core deficits and associated psychiatric comorbidity [4], ASD has been linked to negative non-behavioral health outcomes such as an increased risk of injury [5] and mortality [6,7].

Over time, there has been a significant shift in both the diagnostic criteria and treatment methods for ASD. The majority of current diagnostic procedures involve the use of observational screening instruments that assess a child’s cognitive and social capacities. The 5th edition of the diagnostic and statistical manual of mental disorders (DSM-5) and the modified checklist for autism in toddlers (M-CHAT) are the two most important diagnostic instruments for ASD. These instruments look for long-term problems with social interaction and communication as well as replies to “yes/no” questions that span various areas of development [8].

Multiple lines of evidence indicate that ASD is highly inherited, but environmental factors have also been associated with ASD. Monogenic traits of autism are characterized by a single DNA-level alteration or genetic modification in one gene, independently of environmental factors. These genetic abnormalities are linked to specific syndromes rather than ASD. The development of polygenic traits, conversely, involves a multitude of gene variations combined with environmental influences. As a result, they generally do not produce distinct categories but rather a wide range of phenotypic characteristics that span across a spectrum, influenced by the number and nature of the genes involved and the environmental factors [9,10].

**Figure 1:**
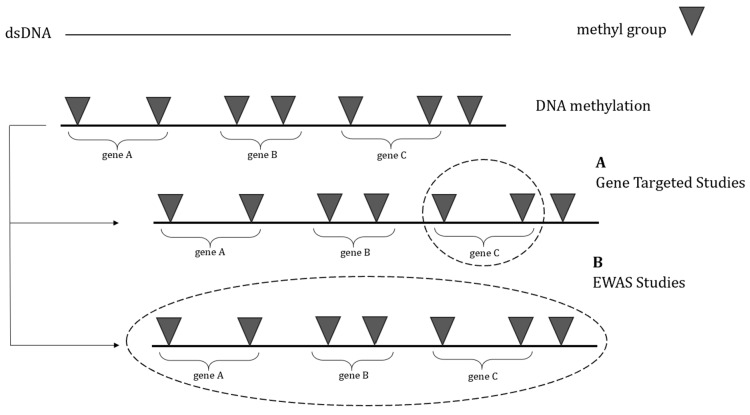
Two approaches in DNA methylation studies. Most of the techniques used in DNA methylation studies can be divided into two main categories: (**A**) gene-targeted studies, which assess methylation at a single specific gene, and (**B**) epigenome-wide association studies (EWASs), which aim to examine genome-wide methylation status.

Extensive genetic research has demonstrated a significant level of genetic variability as the underlying cause of ASD. The initial critical distinction is that ASD can manifest as either an isolated clinical phenotype or as part of a syndromic condition. In the second scenario, numerous phenotypes, including epilepsy, intellectual disability, and dysmorphic characteristics, are present in a developmental disease that manifests as ASD [9,11]. Regarding environmental factors, on the other hand, parental age, maternal medical and mental health, and perinatal environmental exposures have been suggested to play a role in the etiopathogenesis of ASD (Figure 1) [12].

In recent years, there has been a growing interest in examining epigenetic marks in ASD due to their potential mechanistic role in etiology, specifically to explain the effects of environmental exposure or gene–environment interaction associations with ASD or as a biomarker of previous exposure or disease [13]. The expression of many genes is regulated by epigenetic marks that do not change the primary DNA sequence. Both genetic mutations and environmental factors can alter epigenetic modifications, such as DNA methylation, histone methylation, and acetylation. Epigenetic dysregulation has been proven to be linked to autism based on several factors. Primarily, gene mutations involved in epigenetic regulation can cause syndromes associated with autism (Figure 1) [14].

Second, autism was linked to several areas of the chromosome that are influenced by parental imprinting. Repeated reports have shown that individuals with autism have microduplications or microdeletions of the parentally none-printable region 15qllql3 [15,16]. Several studies have indicated an association between ASD and single-nucleotide polymorphisms (SNPs) in a gene directly involved in methylation [17,18]. The expression of the retinoic acid-related orphan receptor alpha gene (*RORA*) and B-cell lymphoma 2 (*BCL-2*) was found to be reduced in the lymphoblastoid cells of autistic patients, demonstrating direct changes in the DNA methylation profile. DNA methylation regulates gene expression patterns by modifying DNA accessibility. From a molecular aspect, when DNA methyltransferases (DNMTs) add a methyl group to the fifth carbon position of cytosine at cytosine–phosphate–guanine (CpG) dinucleotides, DNA methylation takes place [19,20,21,22,23].

In the last two decades, an alarming rise in the prevalence of ASD has intensified the debate over the etiologic roles of non-genetic factors such as epigenetics (primarily abnormal DNA methylation) and environmental interaction [24,25].

This narrative review focuses on the current understanding of how DNA methylation contributes to ASD in patients or animal models. This could be of great assistance for the investigation of the molecular mechanisms underlying ASD, with the ultimate goal of identifying novel targets for the creation of innovative epigenetic drugs.

## 2. DNA Methylation

DNA methylation, the most studied epigenetic modification, is crucially catalyzed by DNA methyltransferases (DNMTs) that transfer a methyl group (CH_3_) from S-adenosyl methionine (SAM) to the fifth carbon of a cytosine residue, resulting in 5mC [26]. It is important to note that DNMT3a and DNMT3b are called de novo DNMT because they can impart a novel methylation pattern onto unmodified DNA. During DNA replication, DNMT1 transfers the DNA methylation pattern from the original DNA strand to the newly formed daughter strand [27]. Moreover, DNMT1 is capable of repairing DNA methylation [28]. DNMT3L, another member of the DNMT family, links with DNMT3a and DNMT3b and stimulates their methyltransferase activity without having the catalytic domain exist in other DNMT enzymes (Figure 1) [29,30,31].

According to the dynamics of the DNA methylation process, its reversibility can occur in two active and passive forms. The passive DNA demethylation takes place in dividing cells as the inhibition or dysfunction of DNMT1 causes newly incorporated cytosine to remain unmethylated, decreasing the overall methylation level after each cell division. Active DNA demethylation is found in both dividing and nondividing cells, but the process requires ten-eleven translocation (TeT) enzymes, which eliminate the methyl group from methylated bases via oxidation [32,33,34].

A significant amount of DNA methylation occurs at CpG sites or cytosines that precede guanine. Due to the mutagenic potential of 5mC, which can deaminate into thymine, mammalian genomes are depleted from CpG sites [35,36].

Except for CpG islands, the remaining CpG sites are widely dispersed and extensively methylated throughout the genome [36]. CpG islands are composed of a minimum of 200 base pairs, have a GC concentration exceeding 15%, and an observed-to-expected CPG ratio exceeding 60%. However, CpG islands and the nearby CpG island shores (2 kb-long regions that reside on both sides of the island) are exceptionally hypomethylated [37,38]. Many of these hypomethylated DNA regions serve as elements that regulate gene expression, including promoters and enhancers [37]. Approximately 70% of gene promoters are located within CpG islands [39]. CpG islands in the promoter regions of actively expressed genes are generally unmethylated, as DNA methylation at promoters is typically associated with gene silencing [26]. Both direct (including steric inhibition of binding with transcription factors) and indirect (using Methyl CpG binding Proteins, MeCPs, that recruit histone deacetylases) mechanisms have been involved in the repression of gene transcription due to CpG island methylation [26,40,41].

Additionally, methylation can occur at non-CpG sites, namely CpA, CpT, and CpC, with CpA being the most commonly methylated. Non-CpG methylation is significantly increased in human embryonic stem cells, neurons, and glial cells [42]. Unlike CpG methylation, which occurs early in development and fails to rise with growth, non-CpG methylation accumulates during neuron development [26,42]. Furthermore, non-CpG methylation increases concurrently with synaptic development and synaptic density. In both human and mouse brain tissue, non-CpG methylation begins during early postnatal development (in the first two years and the first 2–4 months, respectively) and decreases during adolescence. Methylation of non-CpG regions originally parallels the increase in synaptic density that occurs between birth and five years of age in humans but appears to elevate during synaptic pruning through adolescence [43].

This section mainly focused on the DNA methylation catalyzed by DNMTs as a crucial epigenetic modification. This process can be reversed through passive demethylation during cell division and active demethylation involving TeT enzymes. CpG islands, which have high CpG density, are typically hypomethylated and linked to the regulation of gene expression. Non-CpG methylation, particularly CpA, is significant in human embryonic stem cells and neurons, and it increases as synaptic development progresses, reaching its peak during early postnatal development and declining during adolescence.

## 3. Alterations in DNA Methylation at Specific Genes Associated with Autism Spectrum Disorders

Previous researchers exploring the epigenetic alterations in ASD have mainly focused on analyzing specific genes and proteins involved in ASD. Disturbed central and peripheral hemostasis of hormones and neurotransmitters is often found in subjects with ASD [44,45,46,47,48,49,50,51,52,53]. The altered expression of several genes responsible for regulating the hemostasis was reported in ASD patients. Furthermore, DNA methylation in genes associated with the neural matrix [54,55,56,57,58], genes regulating gene expression [55,57,59,60], genes involved in syndromes with a high penetrance of ASD [61,62,63], and genes associated with environmental factors [64,65,66] has recently been recognized in ASD models. Table 1 summarizes the available studies on DNA methylation patterns of ASD patients or animal models. As shown, some paths investigated by several research groups were only preliminarily evaluated, whereas others were widely explored and revealed more consistency between different studies. Clinical data are drawn chiefly from studies in which samples were obtained from human blood cells. In fact, brain tissue analysis is based mainly on animal models. Therefore, despite the limitations of preclinical studies, they could be an essential source of evidence.

The following section provides and compares clinical and preclinical data on DNA methylation at specific genes in ASD patients and animal models. The known clinical impact of different genes led to their classification into various groups.

### 3.1. Hormones and Neurotransmitter Receptor Genes

#### 3.1.1. Serotonin

The serotonin signaling system is implicated in regulating key neurodevelopmental processes [70]. Numerous studies have reported a disruption in serotoninergic neurotransmission in ASD. Patients diagnosed with ASD showed elevated levels of serotonin in the peripheral system but had notably lower levels of serotonin in the central nervous system [71,72]. A variety of ASD symptoms, including insomnia, depression, repetitive behavior, and compulsion, were successfully treated by selective serotonin reuptake inhibitors (SSRIs) [73]. Abnormalities in genes related to serotonin signaling could explain the serotonin depletion in individuals with ASD. DNA methylation in human serotonin receptor 2A (*HTR2A*) and human serotonin receptor 4 (*HTR4*) genes were considered potential candidate genes for ASD.

#####  *HTR2A* 

The etiologic role of three SNPs of *HTR2A* (1438A/G (rs6311), 102T/C (rs6313), and 1354C/T (rs6314)) have been well documented in many neuropsychiatric disorders [74,75]. This gene’s connection to ASD has been investigated in previous studies involving Caucasian, Korean, and Indian populations. While the Korean study found a connection between the haplotype and ASD, the studies on Caucasian and Indian patients failed to identify any association [44,76,77]. However, in the Indian study, the methylation of CpG sites at the 1438A/G and 102T/C loci of the gene was detected. As reported in this study, the HTR2A gene’s promoter lacks CpG islands, which reinforces the gene’s biallelic expression pattern in peripheral blood leukocytes (PBLs). Also, in this study, G and A alleles of 1438A/G (rs6311) polymorphism did not show functional differences [44]. A more recent study by Hranilovic et al. displayed higher mean DNA methylation levels at 1438A/G (regulatory region) of the *HTR2A* gene in autistic subjects than in corresponding controls. However, similar to the previous study on the Indian population, no statistically significant difference was observed between AA and GG carries [45].

#####  *HTR4* 

As reported by recent research on ASD children, hypomethylation of the *HTR4* promoter has been recognized as a possible predictive biomarker for ASD. Additionally, this study indicated the inverse association between age and DNA methylation of the *HTR4* promoter in male ASD subjects [46].

#### 3.1.2. Oxytocin

Oxytocin, a key modulator of socioemotional behaviors, acts via the oxytocin receptor (OXTR), which is enriched in subcortical emotional and reward regions [78]. In humans, it has been observed that resting state functional connectivity between frontal and ventral striatal areas was enhanced by intranasal oxytocin application [79,80]. Pivotal clinical trials demonstrated that intravenous or intranasal administration of oxytocin improves the symptoms of ASD, including repetitive behavior, speech comprehension, and emotion recognition [80,81,82]. In a recent placebo-control randomized trial, four weeks of administration of intranasal oxytocin was found to be associated with a reduction in *OXTR* DNA methylation and enhanced feelings of secure attachment [83]. Studies also evaluated the plasma oxytocin levels in ASD patients and their association with genetic and epigenetic signatures [84,85].

The largest meta-analysis investigating 16 *OXTR* SNPs has found that SNPs rs7632287, rs237887, rs2268491, and rs2254298 were significantly associated with ASD [86]. Also, several reports compared the methylation frequency of *OXTR* between autistic and non-autistic subjects. A research work on the Turkish population showed a significant reduction in the promoter methylation levels in the regions MT1 and MT3 of OXTR, but not in the MT2 and MT4 regions [47]. On the contrary, a study by Andari et al. reported higher *OXTR* methylation frequency in the intron 1 area of MT2 compared to neurotypical controls. Using a combined epigenetic and imaging approach, this study provided the first evidence that *OXTR* hypermethylation is related to the severity of clinical symptoms and brain functional connectivity between cortico-cortical areas participating in the theory of mind. In addition, methylation at a CpG site in the exon 1 area of MT2 has been shown to be positively associated with social responsiveness deficits in ASD [48]. A recent case–control study on twenty adults with high-functioning autism (HFA) revealed no apparent differences in *OXTR* gene sequence, except for rs918316. Also, methylation analysis did not detect a single significantly different methylated site among the 412 CpG sites of the *OXTR* gene in HFA patients compared to normal controls [49].

#### 3.1.3. Dopamine

The intensity and duration of dopaminergic signaling are regulated by the dopamine transporter (DAT) [87]. In various neurodevelopmental disorders, including ASD and attention deficit hyperactivity disorder (ADHD), an altered expression of DAT has been observed [88,89]. In addition, DAT is the target of both psychotropic drugs and neurotoxicants [90,91]. In an earlier animal study, researchers investigated the epigenetic processes that control DAT expression in the midbrain of growing rats. This study showed that mRNA expression of HDAC2, DNMT1, DNMT3a, and DNMT3b decreased with age, while mRNA expression of HDAC5 and HDAC8 increased. This study confirmed that DAT protein and mRNA expression significantly increase with age. It has been mentioned that decreases in *DAT* promoter methylation correlate with the increased gene expression [53].

#### 3.1.4. GABA

The processing of information by neurons and the organization of neural networks depend on maintaining the appropriate balance between excitation (E) and inhibition (I) in the brain [92,93,94]. Many neurological disorders, including ASD, take place when the modulatory mechanisms that control the E/I balance are dysfunctional [95,96]. γ-aminobutyric acid (GABA), an inhibitory neurotransmitter, plays a crucial role in maintaining the E/I balance. *GAD1* is an important gene in regulating GABA levels, and it could be considered an excellent molecular biomarker in assessing E/I imbalances. DNA methylation in the regulatory region of the *GAD1* gene has been compared between ASD patients and healthy controls in a previous clinical study. In the ASD group, it has been reported to exhibit a higher differential methylation pattern and also a greater CpG-wise variance [50].

#### 3.1.5. Insulin

The effects of insulin and insulin-like growth factor 1 (IGF1) are mediated by insulin receptor substrate 2 (IRS2), which is a key downstream component of the signaling cascade [97]. Whole methylome analyses on the human placentas of high-risk pregnancies derived from the MARBLES (Markers of Autism Risk in Babies Learning Early Signs) prospective study identified 400 discriminated differentially methylated regions (DMRs) between children who were later diagnosed with ASD and typically developing controls. One of the reported DMRs is the hypermethylated DMR located at *IRS2*. Also, this study indicated that periconceptional maternal vitamin use modified the methylation at *IRS2* [51].

#### 3.1.6. Estrogen

It has been hypothesized that sex hormones play central roles in ASD etiopathogenesis due to the higher prevalence of ASD in the male population [98]. Increasing lines of evidence have demonstrated that estrogen receptors (ESR1 and ESR2) are involved in reproductive neuroendocrine behavior, memory, learning, and emotion regulation [99,100]. The pathogenesis of several neurologic and psychiatric disorders, including ASD, has been observed to contribute to *ESR* gene expression [52]. ASD patients have significantly reduced ESR2 mRNA and protein levels in their middle frontal gyrus compared with normal individuals [101]. The epigenetic regulation of the *ESR2* gene in a clinical study on Chinese Han males with autism was explored. A relationship between phenotypic features of autism and DNA methylation was investigated. Indeed, in autistic individuals, a total of eight specific CpG sites showed hypermethylation, and four of these sites were found to have a positive correlation with the severity of autistic symptoms [52].

### 3.2. Genes Associated with Neural Matrix

#### 3.2.1. *RELN*

Lintas C. and colleagues evaluated the status of *RELN* gene methylation in post-mortem temporocortical tissue samples from post-pubertal ASD case–control pairs and pre-pubertal ASD individuals. Although the mean percentage of methylation at each CpG position and the mean number of methylated CpGs along the whole *RELN* gene promoter did not differ significantly between ASD and control brains, the distribution of methylated CpG sites was noticeably different. ASD patients had a substantially greater number of methylated CpG islands and heavier methylation in the 5’ region of the *RELN* gene promoter. In contrast, in the 3’ region, the controls exhibited a higher number of methylated CpG positions and a more extensive level of methylation. In ASD brains, only the promoter region located furthest upstream (−458 to −364 bp) showed methylation, whereas in control brains, only the promoter region located furthest downstream (−131 to +1 bp) showed methylation. These results suggest that the pattern of methylation was distinct between ASD and control post-mortem brains. ASD-specific CpG sites in the most proximal gene promoter area might play a functional role by inhibiting *RELN* gene expression, potentially conferring ASD risk. It is important to mention that the findings could be affected by the limited sample size [54].

#### 3.2.2. *SHANK3*

The first study analyzing altered methylation patterns in *SHANK3* in ASD brain samples was published by Li Zhu et al. in 2014 This study involved DNA methylation profiling of five CpG islands (CGI-1 to CGI-5) in the *SHANK3* gene using post-mortem brain tissues from both ASD patients and control subjects. In the cerebellum and cerebral cortex Brodmann region 19 (BA19), they found higher levels of DNA methylation in three intragenic CGIs (CGI-2, CGI-3, and CGI-4). In 15% of ASD brain tissues, elevated methylation was concentrated in CGI-2 and CGI-4. This indicated that *SHANK3* is a useful biomarker for examining the role of gene–environment interaction in the etiology of ASD [55].

Recent investigations on mice exposed to fine particles reported alterations in *SHANK3* methylation along with lower expression levels [102,103,104]. Accordingly, Kang Li and colleagues discovered that exposure to fine particles could result in an autism phenotype in early postnatal rats, which may be a result of the *SHANK3* methylation level and the *SHANK3* signaling pathway. The methylation level at three CpG sites in the *SHANK3* gene from the brain tissues of exposed and control groups was evaluated to explore the epigenetic effects of early postnatal exposure to fine particles on the *SHANK3* gene. At the epigenetic level, the reduced *SHANK3* expression level in juvenile rats exposed to fine particles early in life may have occurred due to an increase in methylation [105].

#### 3.2.3. *ST8SIA2*

Yang X et al. assessed the expression and methylation levels of the *ST8SIA2* gene in the peripheral blood of ASD patients as compared to controls. The ASD group exhibited lower levels of *ST8SIA2* gene expression compared to the control group. Additionally, these expression levels were linked to the severity of ASD in children, as well as their daily life skills, stereotype abnormalities, sensory abnormalities, and self-help ability. ASD children had elevated methylation levels at the *ST8SIA2* gene’s Chr. 15: 92,984,625 and Chr. 15: 92,998,561 sites compared to the control group. The Chr. 15: 92,984,625 site was significantly associated with the stereotypical behaviors of children with ASD [56].

### 3.3. Genes Associated with Gene Expression

#### 3.3.1. *MeCP2*

The methyl-CpG binding protein 2 (MeCP2) gene plays a key role in DNA methylation and transcriptional regulation, with the ability to activate or repress genes such as Brain-derived neurotrophic factor (BDNF) [14]. It is predominantly expressed in mature postnatal neurons, with slight variations across different brain regions, and is crucial for proper neuronal maturation [106] and function [107]. Mutations in *MECP2* lead to Rett syndrome, a severe form of ASD characterized by impaired social behaviors, cognition, and coordination [108]. Despite the well-established role of *MECP2* in Rett syndrome and occasional instances in autism [109], there is a lack of extensive research investigating the relationship between DNA methylation and *MECP2* expression in samples of individuals with ASD.

The only clinical study examining the correlation between DNA methylation and the expression rate of the *MeCP2* gene in brain samples from people with ASD was carried out by Nagarajan and colleagues in 2006. To achieve this, samples were collected from 14 frontal cortex samples from ASD patients, with six fusiform gyrus samples also being obtained. As expected, a significant reduction in *MeCP2* expression was found in the experimental group. Further tests were performed to measure the level of DNA methylation across the *MeCP2* gene. The promoter region, particularly at CpG site #3, showed a remarkable increase in the methylation level in cells with reduced gene expression. This site is well known as a binding target for transcription factors. However, this observation was more apparent in juvenile autism samples than in adult ones, suggesting that other indirect factors impact *MeCP2* activation as the individual ages. In this study, the limited number of participants prevents making generalizations to larger populations [59].

In light of the putative role of the *MeCP2* gene in ASD pathogenesis, the alteration in its expression served as a key metric for Zhou et al. [58] to evaluate the impact of air pollution (nitrogen dioxide and fine particles) on animal brains. Healthy rats were exposed to varying levels of air pollutants throughout full gestation and 12 days postnatal, after which brain samples were collected. The results revealed that rats residing in areas with higher pollution levels exhibited reduced instances of social preference and novelty (behaviors commonly associated with ASD), decreased MeCP2 content, and elevated methylation of the gene. These changes were particularly pronounced in rats exposed during postnatal days, highlighting the heightened sensitivity of DNA methylation to early-life insults. Also, in a recent study, researchers employed locus-specific epigenetic modulation to induce the methylation of the transcription start site (TTS) of *MeCP2* in healthy male rats. As a result, there was a significant decrease in MeCP2 content in the hippocampus and, to a certain extent, in the parietal cortex. The alterations resulted in the development of behaviors resembling ASD in the rats, such as reduced sociability, more extended periods of self-grooming, poor memory function, and anxiety/depression. This observation places further emphasis on the causal connection between the methylation of the *MeCP2* gene and disease symptoms [64].

#### 3.3.2. *NHIP*

Using cutting-edge techniques to evaluate the methylome profile of genes, Zhu et al. [60] have introduced a gene formerly overlooked by prior studies on the genome of individuals with ASD. The gene in question, *NHIP* (neuronal hypoxia-inducible, placenta-associated), regulates other genes and optimizes neural cells’ response to environmental insults such as hypoxia and oxidative stress. *NHIP* expression at elevated levels acts as a protective factor, preventing neurodevelopmental defects. This explains the reduced content of *NHIP* in 46 placentae from ASD children in this study. However, this locus was found to be hypo-methylated instead of the usual association of higher methylation with lower gene expression. The authors interpret this as a reflection of previously or currently low levels of *NHIP*. Interestingly, early supplemental therapy with folic acid reversed the reduced *NHIP* levels and methylation, presenting *NHIP* as a promising therapeutic target to be considered in future studies. Given that this is the first and only ASD study involving this gene, and since it was carried out using whole-genome sequencing, further research focused on the link between *NHIP* expression, methylation, and ASD symptoms is warranted to draw an accurate conclusion.

### 3.4. Genes Involved in Disorders Associated with ASD

#### 3.4.1. *FMR1*

Fragile X syndrome (FXS) is the leading monogenic cause of ASD. The most common cause of FXS is silencing of the fragile X mental retardation 1 (*FMR1*) gene by DNA methylation and aberrant heterochromatinization. Subsequently, the absence of the FMR1 protein (FMRP) leads to the FXS phenotype and autism [110,111]. In a clinical study on twelve males with FXS and atypical mosaicism, decreased expression levels of *FMR1* mRNA and FMRP were observed in half of the study population. These reductions were correlated with the presence of ASD and lower IQ as well as the extent of DNA methylation [61]. The results align with those reported by Budimirovic et al., which confirmed the inverse association between a decrease in FMRP levels and the overall severity of the FXS phenotype. Also, two-fold lower FMRP levels were reported in FXS with ASD compared to FXS without ASD [62].

#### 3.4.2. *APOE*

Abnormal methylation of the apolipoprotein E (*APOE*) gene has been found to be associated with Alzheimer’s disease, which might have overlapping mechanisms with ASD [112,113]. *APOE* hypermethylation has been observed in the peripheral blood DNA of pediatric patients with ASD. It has been documented that the percentage of methylation of a reference (PMR) of 15.4% was the most effective threshold for predicting ASD [63].

### 3.5. Genes Associated with Environmental Factors

#### 3.5.1. *WNT*

Prenatal exposure to valproic acid, a medication used for mood stabilization and epilepsy treatment, has been shown to elevate the risk of ASD [114,115]. Preclinical studies have reported that ASD-like behaviors, including decreased socialization and activated stereotyping, were induced by the administration of VPA in pregnant rodents [116,117].

A preclinical study by Wang et al. revealed that rat exposure to VPA in early pregnancy induced demethylation in the promoter regions of *WNT1* and *WNT2* genes, thus increasing the mRNA and protein expression of WNT1 and WNT2 in the hippocampi and prefrontal cortexes of the offspring. This induced dysregulation in the WNT/b-catenin signaling pathway facilitates susceptibility to ASD [65].

#### 3.5.2. *KCC2*

Several studies suggested that exposure to general anesthesia (GA) during cesarean section (CS) was associated with susceptibility to ASD [118]. However, a recent study did not support the role of GA in enhancing the risk of ASD [119].

The expression of the hypothalamic Kþ-2ClClexporter (*KCC2*) gene has been indicated to be decreased in a rat model exposed prenatally to sevoflurane, the most common anesthetic in pediatrics. This study reported increased methylation in the *KCC2* promoter, concordant with the alterations in *KCC2* expression. Another finding was that females were at a diminished risk compared to males of developing abnormalities in behavioral testing, memory, and the expression of *KCC2* [69].

### 3.6. Other Genes

#### 3.6.1. *ACSF3*

Recent research on Saudi autistic children found no DNA methylation in the specificity protein 1 (SP1) binding site within the Acyl-CoA synthetase family member 3 (*ACSF3*) promoter. However, a notable connection was reported between the gene expression of SP1 and *ACSF3* in ASD patients [67].

#### 3.6.2. *PPP2R2C*

Kimura et al. demonstrated that the *PPP2R2C* gene was downregulated and hypermethylated in ASD patients compared to the control group [68].

#### 3.6.3. *CYP2E1*

An analysis of placentas from the MARBLES study showed hypomethylated DMR at the *CYP2E1* gene, which is associated with ASD diagnosis (Table 2) [51].

To conclude this section, we categorized the ASD-associated genes into various groups based on their clinical impact and discussed the current knowledge about the connection between their methylation and ASD. It is worth noting that, according to a recent meta-analysis, the phenotypical features of autism could be associated with the methylation of specific genes. For example, a connection between methylation in the *ST8SIA2* gene and behavioral phenotypes of ASD has been reported. Also, severe forms of ASD showed higher methylation in the *RELN* gene [136].

## 4. Global DNA Methylation Profiling in Autism Spectrum Disorder

In recent years, due to advances in DNA methylation analysis for various applications, epigenome-wide association studies (EWASs) have become more common for assessing the relation between epigenetic alterations and disease occurrence by representing genome-wide methylation profiles.

The primary method mentioned in most epigenome-wide association studies involves assessing genome-wide DNA methylation using the Infinium HumanMethylation450K BeadChip array technology.

The fundamental benefit of global DNA methylation profiling over targeted DNA methylation studies is the ability to observe the full picture of the modifications connected to the pathology rather than just concentrating on individual genes of interest. As a result, this method enables the identification of novel potential pathways related to ASD pathophysiology mechanisms.

In this section, studies using an epigenome-wide approach method will be discussed. The studies will highlight changes in DNA methylation profiles linked to ASD or cognitive problems and repetitive behaviors in animal models. The majority of the information is derived from clinical research, which is described below. Details from the EWASs are shown in Table 2.

### 4.1. Clinical Evidence

This review divides clinical studies examining genome-wide DNA methylation alterations into two groups.

In the first group, the researchers assessed the DNA methylation profile in post-mortem brain tissues, while in the second group, the EWAS was conducted on peripheral tissues. In both groups, some important regions in the genome associated with ASD mechanism were found to be epigenetically altered, which suggests some new evidence for the relevance of autism and DNA methylation changes and can predict some novel insights for therapeutic approaches.

#### 4.1.1. EWASs on Post-Mortem Brain Tissues

Ladd-Acosta C et al., in 2014, conducted a study to report the first genome-wide examination of DNA methylation outside of CpG islands and promoters in ASD among three brain regions of PFC, TC, and CBL [120]. This study compared the DNA methylation between 19 ASD cases and 21 controls of post-mortem brain samples using the Infinium Human Methylation 450 BeadChip (450K). They also adopted the “bump hunting” method to detect differentially methylated regions (DMRs) more effectively. The results showed four significant (adjusted *p* < 0.1) DMRs across the genome, with three DMRs found in the superior TC and one in the CBL. The DMR at the site of 3′ UTR of proline-rich transmembrane protein 1 (PRRT1), as well as DMRs within the promoter regions of tetraspanin 32 (*TSPAN32*) and chromosome 11 open reading frame 21 (*C11orf21*) were less methylated in the TC of the ASD cases in comparison to controls. Also, they identified a hypermethylated DMR in the TC of autistic cases, located in an intergenic region, with the nearest gene being *ZFP57*. Only one significant DMR located within *SDHAP3* was identified in CBL samples, which was hypermethylated in ASD cases.

A study published in 2014 by Nardone S et al. investigated genome-wide DNA methylation patterns in 46 brain samples of 13 ASD cases and 12 controls in two brain areas, the prefrontal cortex (Brodmann Area 10; BA10) and the anterior cingulate gyrus (Brodmann Area 24; BA24) and also the significance of dysregulation in DNA methylation in the development of the disorder [121]. In this study, the Illumina 450 K methylation array identified many dysregulated CpGs in these two cortical regions of brains from ASD patients. The results revealed 5329 CpG sites that exhibited differential methylation patterns between control and autism cohorts in BA10 and 10,745 in BA24. Also, the researchers indicated that many immune response-associated genes were hypomethylated in autistic samples, and overexpression of these genes demonstrated the significant role of epigenetic alterations in the dysregulation of the immune system as an involving factor in ASD.

Nardone S and colleagues 2017 conducted another genome-wide methylation study on fluorescence-activated cell sorting-sorted neuronal nuclei from the anterior prefrontal cortex (PFC), BA10, BA9, and BA8 to assess the changes in DNA methylation patterns in cortical neurons of individuals with ASD [122]. In this study, by using next-generation bisulfite sequencing (NGBS), they validated 37 CpGs across 4 DMRs and showed a strong correlation between the methylation values detected by 450 K Bead Array and targeted NGBS. DMRs related to gamma-aminobutyric acid type B receptor subunit 1 (*GABBR1*) and Mir124-2 showed hypomethylation, and two other DMRs associated with family with sequence similarity 124 member B (*FAM124B*) and long non-coding RNA nuclear enriched abundant transcript 1 (*lnNEAT1*) were hypermethylated in ASD versus the control group. There is an association between *Mir124* and social behavior, which has been mentioned in several studies [137,138].

Another genome-wide methylome analysis study published in 2019 detected significant DNA methylation defects in the subventricular zone of the lateral ventricles from the post-mortem brain of 17 ASD versus TD individuals [123]. The authors discovered significant differences (*p* < 0.05) in DNA methylation levels in certain genes. These genes are dynamic genomic regions, presumed to be involved in transcriptional regulation due to specific histone modification states. These differences were found between ASD cases and controls in the CpG sites from the 450k array dataset. They also expressed that 5-methylcytosine (5-mC) in ASD brain was hypomethylated at transcription start sites (TSSs), gene bodies, and canonical exons in comparison to controls, which confirmed the overall hypomethylation detected in young ASD cases from the 5-mC ELISA assay.

In 2019, Wong C et al. extracted and analyzed 223 post-mortem tissue samples from 43 individuals with ASD and 38 control donors to determine genome-wide patterns of DNA methylation in three brain areas of PFC, TC, and CB by using a Illumina Infinium Human Methylation-450 BeadChip array of bisulfite converted DNA [124]. The authors expressed that many co-methylated modules strongly related to ASD were found by cortical co-methylation network analysis, and these modules were shown to be enriched for genomic regions assigned to genes involved in immune system function, synaptic signaling, and neuronal regulation.

A recent case study conducted by Takahashi E and colleagues in 2022 showed the relationship between ASD and epigenetic analyses by using MRI and diffusion tractography, whole-genome bisulfite sequencing (WGBS), flow cytometry, and RT qPCR [125]. The findings revealed that there were distinct methylation patterns in the components of three important networks associated with ASD. This was discovered through an IPA analysis of WGBS data from samples of the dorsal insula region of the brain. These networks are involved in neurological diseases, developmental disorders, as well as nervous system development and function.

#### 4.1.2. EWASs on Peripheral Tissues

##### Studies on Monozygotic Twin Pairs

To the best of our knowledge, three clinical studies reported genome-wide DNA methylation alteration in monozygotic twin pairs with autism to show the relevance of ASD and epigenetic alterations. Wong C et al., in 2014, investigated the impact of environmentally driven epigenetic factors on ASD by conducting a genome-wide analysis of DNA methylation in a cohort of 50 pairs of monozygotic twins [126]. Numerous differential methylation areas linked to ASD were discovered using within-twin and between-group analysis. Their results also showed significant correlations between DNA methylation and autistic trait scores.

In another study in 2019, Liang S et al. investigated the contribution of DNA methylation to ASD etiology in discordant monozygotic twins [127]. The study involved the genome-wide analysis of DNA methylation using samples obtained from five pairs of monozygotic twins discordant for ASD. Their results showed a total of 2397 differentially methylated genes. Additionally, this gene list’s annotations in the Kyoto Encyclopedia of Genes and Genomes showed that monozygotic twins with ASD predominantly activated the neurotrophin signaling pathway. By using bisulfite-pyrosequencing, the methylation of the SH2B1 gene was further validated in monozygotic twins with ASD-discordant or ASD-concordant traits as well as a group of 30 pairs of sporadic case–control cases. The findings demonstrated that ASD-discordant monozygotic twins had a more considerable DNA methylation difference than ASD-concordant monozygotic twins. Additionally, the SH2B1 Chr.16:28856743 analysis revealed notable variations in DNA methylation between the case and control groups. These findings imply that the etiology of ASD is related to abnormal SH2B1 methylation.

##### Studies on ASD Patients

Hanon E et al., in 2018, was the first study to examine prenatal epigenetic variation linked to autism quantified neonatal methylomic alteration in 1263 infants with a 1:1 ratio of ASD and TD individuals [129]. Neonatal blood samples were obtained proximal to birth, and then the authors developed an enormous database of DNA methylation quantitative trait loci (mQTL), which were utilized to define the molecular implications of genetic variations linked with ASD. The results of this study demonstrated that neonatal DNA methylation did not significantly differ in ASD individuals in comparison to controls; however, methylomic variation at certain loci in blood at birth was revealed to be related to an elevated polygenic burden for autism. Another study published in 2022 assessed the global DNA methylation profiles of peripheral blood samples of 23 ASD cases, 23 cases of FXS with ASD (FXSA), and 11 TD children by using the Human Methylation EPIC Bead Chip [133]. They found out DNA methylation alteration has a key role in the pathology of these disorders by characterizing the methylome profile of each group.

Zhu Y and colleagues (2019) investigated the role of epigenetics in ASD occurrence risk by using a novel strategy to detect differentially methylated regions (DMRs) in complete methylomes from placenta samples of male children with ASD compared to the control group [51]. The researchers in this study discovered new methylation alterations at the CYP2E1 and IRS2 genes, which showed genome-wide significant differences between ASD and the control group. Both the CYP2E1 and IRS2 DMRs may be found in situations close to the transcription start site (TSS) in CpG shore intragenic regions. This finding is consistent with enriching TSS flanking regions and H3K4me3 promoter marks in the 400 ASD DMRs. This study has also shown that both of these genes are involved in protein synthesis, cell proliferation, and cell metabolism, which is the same as the results of similar studies of gene pathways in ASD showed. In 2020, Mordaunt C et al. conducted a whole-genome bisulfite sequencing of 152 umbilical cord blood samples to exhibit the association of DNA methylation and ASD at birth [130]. The autosomal ASD DMRs detected in this study were shown to be highly enriched for promoter and bivalent chromatin states in the majority of cell types. Moreover, the binding sites of methyl-sensitive transcription factors, which are important in developing the brain, were significantly found in those DMRs identified in cord blood.

Bahado-Singh R and colleagues 2021 published a study about genome-wide methylation in the placental tissue of ASD patients using the Illumina 450K array and artificial intelligence for analysis of differentially methylated loci [131]. The results showed that full-term birth ASD patients had 9655 CpGs with significantly altered methylation. Additionally, 2802 CpGs were intergenic markers, and 6853 (4129 genes) were intragenic. They found 3820 CpGs that were hypomethylated and 3033 that were hypermethylated for intragenic CpG markers. The quantity of synapse, microtubule dynamics, neuritogenesis, and abnormal morphology of neurons are four biological functions that were significantly overexpressed in ASD cases compared to controls.

Mordaunt C et al. conducted another study in 2022 about comethyl, which was a network-based methylome approach to investigate the correlation between DNA methylation and autism [132]. The researchers reported that differentially methylated genes that had important roles in developing brain and glial functions were detected in their sequencing-based analysis of cord blood samples from autism cases in comparison to controls. And at last, in 2022, Zhu Y and colleagues performed a WGBS in the placenta to investigate a previously indeterminant ASD risk gene, *LOC105373085*, renamed *NHIP* [60]. The results showed that *NHIP*, which had a role in cell proliferation, regulating synapses and neurogenesis directly and indirectly, was hypomethylated and had significantly increased expression in ASD vs. controls.

### 4.2. Preclinical Evidence

Only two preclinical studies have examined the DNA methylation changes in autism in a genome-wide aspect. In 2015, Papale L et al. assessed a genome-wide map of striatal 5-hydroxymethylcytosine (5hmC) in an autism mouse model by using chemical labeling and an affinity purification method coupled with high-throughput sequencing technology [134]. 5hmC, as an altered DNA type, is dramatically found in post-mitotic neurons and is relevant to the active transcription of neuronal genes. The results showed many differentially hydroxymethylated regions (DHMRs) in the genome profile, which were significantly located in some known ASD-associated genes. This study guided us to understand the role of 5hmC in ASD and suggest some novel ASD-associated genes. Another preclinical study published by Muehlmann A et al. in 2019 examined the hypothesis that a methyl donor-supplemented diet has a role in the occurrence of ASD in C58 mice by modifying DNA methylation [135]. The results showed that exposure to this diet affects DNA methylation increases in the brain tissues of the autistic group, which is significantly related to the frequency and patterns of repetitive behavior in the mouse model of autism.

Taken together, based on clinical studies on genome-wide DNA methylation alterations in autism, evidence of epigenetic changes was found in both post-mortem brain tissues and peripheral tissues. DMRs in genes associated with ASD were identified in various studies on post-mortem brain tissues, suggesting a potential connection between autism and DNA methylation changes [120,121,122,123,124,125]. These findings could offer new perspectives for therapeutic approaches. In other ways, conducting EWASs on peripheral tissues demonstrated that several studies have investigated DNA methylation alterations in twins with autism, showing correlations between DNA methylation and autistic traits and differential gene expression in ASD-discordant twins [126,127,128]. Additionally, studies on ASD patients revealed that neonatal DNA methylation variations are related to an elevated polygenic burden for autism, and DNA methylation alterations play a key role in the pathology of ASD and related disorders [51,60,129,130,131,132,133]. Two preclinical studies have examined DNA methylation changes in autism on a genome-wide level. One study found altered DNA type 5hmC in an autism mouse model, suggesting some novel ASD-associated genes [134]. Another study suggested that a diet supplemented with methyl donors may affect DNA methylation in the brain tissues of autistic mice, influencing repetitive behavior patterns [135]. Specific details from these studies can be found in Table 2.

## 5. Conclusions

This review collates the existing literature on the association between DNA methylation and ASD. Although it is generally agreed that changes in DNA methylation patterns are associated with ASD phenotype and pathology, the potential use of DNA methylation as a predictive or diagnostic biomarker for ASD is still being debated. Given the limited number of studies and the inconsistency between their findings, DNA methylation in ASD requires more thorough investigation. As represented in Table 1, DNA methylation alterations at certain genes show apparent discrepancies among studies. In other words, the available information did not concur to determine whether DNA hypomethylation or hypermethylation within each particular gene is associated with ASD. Although transcriptional expression studies currently offer valuable insights into the significance of epigenetic changes in the clinical evaluation of ASD, the available data on DNA methylation in each specific gene were limited to only one or two studies. However, in more detail, methylation at HTR2A, OXTR, and MeCP was more extensively evaluated.

Considering the growing recognition of epigenetic alterations associated with ASD, identifying the most impactful epigenetic profiles will enable researchers to concentrate their studies on the genes most likely to be relevant to disease pathogenesis. Our narrative review highlights the significance of DNA methylation among epigenetic changes. However, based on the papers reviewed here, current knowledge is insufficient to determine of which specific gene’s methylation could be used as a biomarker or drug target.

In EWAS approaches, which have the ability to identify the potential mechanisms associated with ASD pathophysiology, the full picture of modifications was manifested rather than only focusing on a single specific gene. Here, genome-wide research on post-mortem brain tissues as well as peripheral tissues suggests the significant relevance of DNA methylation in some important regions of the genome with ASD. However, further meta-analyses and more extensive controlled trials are required regarding EWAS limitations, including various techniques and statistical analysis approaches.

Several issues should be taken into consideration before accepting any final conclusion: (1) Although most of the clinical studies presented here were performed on children and the male population, significant confounding effects could be expected from differences in race, ASD diagnostic criteria, sample size, and technical perspectives. (2) Different studies concentrated on various DNA methylation sites within a single gene, which need to be highlighted. For instance, the methylation of CpG sites at 1438A/G and 102T/C loci of the HTR2A gene, MT1, and MT3 regions of the OXTR gene were proposed as candidate diagnostic biomarkers [44,47]. (3) Investigations on the post-mortem brain are the most reliable studies for comprehending the pathological processes taking place in the brains of ASD patients, which was not feasible in the majority of the clinical studies examined in this review. (4) Indeed, despite the limitations of animal studies, the mechanistic link between alternation in DNA methylation at specific genes, brain area, and behavior is taken chiefly from preclinical evidence, which is still scarce.

Eventually, in recent years, there was a rise in studies focusing on DNA methylation to understand the epigenetic mechanisms related to ASD. Clinical studies on peripheral DNA have identified specific DNA methylation markers linked to ASD. Nonetheless, further studies on animal models would aid in realizing etiopathogenetic processes involved in ASD and also in identifying putative molecular targets for innovative therapeutic approaches.

## Figures and Tables

**Table 1:** Studies reported differences in DNA methylation at specific genes in ASD patients or animal models of ASD.

	Clinical Studies								
Gene	Protein	Genomic Region	Groups of Subjects	Tissue	ASD Diagnosis	N of Subjects (in Each Group)	Age of Subjects (in Each Group)	Main DNA Methylation Finding	Ref.
*HTR2A*		Promoter	ASD, HC	Blood	DSM-4	90 + 101	5.84 ± 2.96 y	No association was proved	[44]
		Promoter	ASC, HC	Blood	DSM-4	90 + 66	4–45 y 19–60 y	Different methylation patterns	[45]
*HTR4*		Promoter	ASD, HC	Blood	DSM-5	61 + 66	4.02 ± 2.83 y 5.76 ± 0.72 y	↓ in ASD and females	[46]
OXTR	Oxytocin receptor	Promoter	ASD, TD	Blood	DSM-5-TR, CARS	27 + 39	39.27± 16.95 m 43.69 ± 20.51 m	↓ in ASD	[47]
	Oxytocin receptor	Promoter	ASD, neurotypical group	Saliva	DSM-5 ASD	35 + 64	27.02 ± 5.34 y 28.22 ± 6.96 y	↑ in the intron 1 area in ASD	[48]
	Oxytocin receptor	Promoter	HFA, HC	Blood	German guideline	20 + 20	30.5 ± 7.8 y 30.7 ± 7.8 y	Not reported a single significant group-dependent methylated site	[49]
*GAD1*	Glutamic acid decarboxylase	Regulatory	ASD, control	Brain	DSM-5	4 + 5	25.9 ± 0.8 y 26.1 ± 0.8 y	More diverse in cerebral organoids from ASD subjects	[50]
*IRS2*	Insulin receptor substrate 2	Exon 1, Intron 1	ASD, TD	MARBLES placenta	DSM-5	20 + 21	-	↑ in ASD	[51]
*ESR2*	ESR2	Proximal promoter region and an untranslated exon	ASD, HC	Blood	DSM-4	54 + 54	4.24 ± 0.98 y 4.37 ± 0.80 y	Different methylation patterns	[52]
*RELN*	Reelin	Promoter	ASD, HC	Brain	-	6 + 6	21.0 ± 2.9 y 22.0 ± 1.8 y	Different methylation patterns	[54]
*SHANK3*	SH3 and multiple ankyrin repeat domains 3	Promoter	ASD, control	Brain	DSM-5 and ADI-R	54 + 43	-	↑ SHANK3 CGIs	[55]
*ST8SIA2*	ST8 alpha-N-acetyl-neuraminide	Chr. 15: 92,984,625/Chr. 15: 92998561	ASD, TD	Blood	DSM-5, ADI-R, ADOS	69 + 76	4.47 ± 1.23 y 4.59 ± 1.19 y	↑ in ASD	[56]
*MeCP2*	Methyl CpG binding protein 2	Promoter	ASD, control	Brain	Not mentioned	9 + 9	5–56 y 9–56 y	↑ in ASD	[59]
*NHIP*	Neuronal hypoxia-inducible, placenta associated	22q13.33	ASD, TD	EARLI/MARBLES placenta	ADOS/ADI-R/MSEL	MARBLES: 46 + 46 EARLI: 16 + 31	-	↓ in ASD	[60]
*FMR1*	FMRP	Promoter	FXS, control	Blood	ADOS	12 + 5	1–28 y 1–28 y	↑ in 4 cases (3 of which were ASD)	[61]
		Gene	FXS + ASD, HC	Blood	DSM-5	18	Male: 14.4 ±11.9 y Female: 14.7 ± 10.9 y	FMRP levels correlate with FMR1 gene methylation	[62]
*APOE*	APOE	Promoter	ASD, HC	Blood	DSM-4	62 + 73	-	↑ in ASD	[63]
*ACSF3*	Malonyl-CoA synthetase	Promoter	ASD, control	Blood	DSM-5	19 +18	6–12 y 3–11 y	No DNA in the binding site of SP1 within the ACSF3 promoter	[67]
*PPP2R2C*	PP2A	Promoter	ASD, HC	Blood	DSM-5, ADOS ASSQ-R	29 + 29	-	Hypermethylation and gene downregulation	[68]
*CYP2E1*	Cytochrome P450 Family 2 Subfamily E Member 1	Intron 1, Exon 2	ASD, TD	MARBLES placenta	DSM-5	20 + 21	-	↓ in ASD	[51]
**Preclinical Studies**									
**Gene**	**Protein**	**Genomic Region**	**Animal/Strain**		**Animal Model**	**Tissue**	**Age of subjects**	**Main DNA Methylation Finding**	**Ref** **.**
*DAT*	DAT	Promoter	Long-Evans rat			Midbrain and striatum	10 w	↑ in midbrain and striatum	[53]
*RELN*	Reelin	Promoter	Balb/cAnNCrlBR mice			Cerebral cortex	4 d	Unmethylated	[57]
*SHANK3*	SH3 and multiple ankyrin repeat domains 3	Promoter	Wistar rats		PND exposure to pollution	Brain	25 d	↑ in exposed rats	[58]
*MeCP2*	MeCP2	TSS	Adult mice			Hippocampus	3–18 w	↑ in hippocampus	[64]
	methyl CpG binding protein 2	Promoter	Wistar rats		PND exposure to pollution	Brain	25 d	↑ in exposed rats	[58]
*WNT*	WNT1/WNT2	Promoter	Rat		Rat VPA ASD model	Frontal cortex and hippocampus	Offspring	Increased expression	[65]
*KCC2*	Potassium (K^+^)/chloride (Cl^−^) symporter	Promoter	Sprague Dawley rats		Parental exposure to sevoflurane	Hypothalamus, hippocampus	5 d	↑ only in male offspring	[69]

Abbreviations: ASD: autism spectrum disorder; TD: typical development; HC: healthy control; HFA: high-functioning autism; HTR2A: human serotonin receptor2A; DSM-4: Diagnostic and Statistical Manual of Mental Disorders, fourth edition; ASC: Autism Spectrum Condition; HTR4: human serotonin receptor 4; DSM-5-TR: Diagnostic and Statistical Manual of Mental Disorders, Fifth Edition, Text Revision; CARS: Childhood Autism Rating Scale; OXTR: oxytocin receptor; GAD1: Glutamic acid decarboxylase 1; DAT: dopamine transporter; IRS2: insulin receptor substrate 2; ESR2: estrogen receptor beta; CGIs: CpG Islands; ST8SIA2: ST8 alpha-N-acetyl-neuraminide alpha-2,8-sialyltransferase 2; MeCP2: methyl-CpG binding protein 2; NHIP: neuronal hypoxia-inducible, placenta associated; APOE: apolipoprotein E; KCC2: Kþ-2ClClexporter; ADOS: Autism Diagnostic Observation Schedule; MSEL: Mullen Scales of Early Learning; ADI-R: Autism Diagnostic Interview—Revised; SP1: specificity protein 1; ACSF3: Acyl-CoA synthetase family member 3; PP2A: Protein Phosphatase type 2 A; CYP2E1: cytochrome p450 2E1; EARLI: The Early Autism Risk Longitudinal Investigation; MARBLES: Markers of Autism Risk in Babies Learning Early Signs; FMR1: fragile X mental retardation 1; FMRP: fragile X mental retardation protein; FXR: fragile X syndrome; SHANK3: SH3 and multiple ankyrin repeat domains 3; PND: Postnatal day; VPA: Valproate. y: years, m: months, w: weeks; d: days, ↑: increase, ↓: decrease.

**Table 2:** EWASs on ASD patients or animal models of ASD.

Clinical Studies					
Subjects	ASD Diagnosis	Tissue	N of Subjects	Methods	Refs.
**Autism cases vs. unrelated controls**	ADI-R, ADOS	Post-mortem brain tissue (dorsolateral prefrontal cortex, temporal cortex, and cerebellum)	19 autism cases, 21 unrelated controls	Infinium HumanMethylation450 BeadChip, bump hunting approach, and a permutation-based multiple testing correction method	[120]
**autism cases vs. controls**	ADI-R	Two cortical regions, prefrontal cortex (BA10), and anterior cingulate gyrus (BA24)	13 autism cases, 12 controls	DNA was converted with sodium bisulfite and probed with the Illumina 450 K methylation array	[121]
**ASD vs. controls**	ADI-R	Anterior PFC, BA10, frontal cortex BA9 and BA8	16 male ASD and 15 male controls	450 K BeadArray, targeted next-generation bisulfite sequencing	[122]
**ASD vs. TD**	ADI-R	Subventricular zone of the lateral ventricles	17 ASD, 17 TD	ELISA, Illumina 450k Array-Based DNA Methylation Analyses	[123]
**ASD vs. non-psychiatric control donors**	Not mentioned	Post-mortem tissues samples [PFC, TC, and CBL]	43 ASD, 38 controls	Illumina Infinium HumanMethylation450 BeadChip array of bisulfite converted DNA	[124]
**Case study** **ASD**	ADI-R, ADOS	Post-mortem brain samples, SVZ, and insular cortex	7 cases	Whole-genome Bisulfite Sequencing	[125]
**MZ twins discordant**	Not mentioned	Blood	50 MZ twin pairs	Illumina Infinium HumanMethylation27 BeadChip array of bisulfite-converted DNA	[126]
**ASD Monozygotic Twins**	DSM-5, ADOS	Blood	5 pairs of ASD-discordant MZ twins, 4 pairs of ASD-concordant MZ twins, and 30 pairs of sporadic patients	Illumina Infinium Human Methylation 450BeadChip array of bisulfite converted DNA	[127]
**MZ twins; concordant ASC vs. discordant ASC vs. control**	ADI-R, ADOS	Blood	6 concordant ASCs, 6 discordant ASCs, and 11 control pairs (total N = 46)	Illumina 27 K DNA methylation dataset; the edgeR package for differential expression analyses	[128]
**ASD cases vs. matched controls**	DPCRR, DNPR	Blood	1316 (equal numbers of ASD cases and matched controls)	mQTL	[129]
**MARBLES** **ASD vs. TD**	ADOS, ADI-R, MSEL, DSM-5	Placenta	20 ASD, 21 TD	Illumina HiSeq 2000 array of bisulfite converted DNA	[51]
**TD vs. ASD**	ADOS, MSEL	Umbilical cord blood samples	56 TD, 50 ASD	Whole-genome bisulfite sequencing	[130]
**Autism cases vs. controls**	DSM-IV	Placental tissue	14 cases, 10 control	Illumina HumanMethylation450 BeadChip (450K)	[131]
**TD vs. ASD**	ADOS, MSEL	Male cord blood samples	39 TD, 35 ASD	Develop the R package Comethyl	[132]
**ASD vs. TD (MARBLES and EARLI)**	ADOS, ADI-R, MSEL, DSM-5	Placenta	46 ASD, 46 TD	WGBS, Illumina HiSeq X array of bisulfite converted DNA	[60]
**ASD, FXSA, TD**	ADOS, DSM-V	Peripheral blood	23 ASD, 23 FXSA, 11 TD	Data processing and analysis were performed in R (version 4.0.2) using minfi, limma, DMRcate, ChAMP, and methylCC packages	[133]
**Preclinical Studies**					
**Animal/Strain**	**Animal Model**		**Tissue**	**Method**	**Refs.**
**Mice**	autism mouse model (*Cntnap2^−/−^*)		striatum	Bioconductor package edgeR	[134]
**Mice**	C58 mice		cerebellum, striatum, and cortex	MethylFlash Methylated DNA Quantification Kit (Epigentek) protocol	[135]

Abbreviations: TD: typically developing; ADOS: Autism Diagnostic Observation Schedule; MSEL: Mullen Scales of Early Learning; ADI-R: Autism Diagnostic Interview—Revised; BA10: Brodmann area 10; BA24: Brodmann area 24; PFC: prefrontal cortex; WGBS: whole-genome bisulfite sequencing; ASC: Autism Spectrum Condition; mQTL: DNA methylation quantitative trait loci; DPCRR: Danish Psychiatric Central Research Register; DNPR: Danish National Patient Register; ADOS-2: Autism Diagnostic Observation Schedule, Second Version; PFC: prefrontal cortex; TC: temporal cortex; CBL: cerebellum; FXSA: Fragile X syndrome with ASD; MZ: Monozygotic; Dup7: 7q11.23 duplication syndrome; SVZ: subventricular zone.
